# Filtering Strategies for Deformation-Rate Distributed Acoustic Sensing

**DOI:** 10.3390/s22228777

**Published:** 2022-11-14

**Authors:** Jihyun Yang, Jeffrey Shragge, Ge Jin

**Affiliations:** 1 Center for Wave Phenomena, Department of Geophysics, Colorado School of Mines, 1500 Illinois St., Golden, CO 80401, USA; 2 Reservoir Characterization Project, Department of Geophysics, Colorado School of Mines, 1500 Illinois St., Golden, CO 80401, USA

**Keywords:** fiber optic, interrogator units, distributed acoustic sensing, filter design

## Abstract

Deformation-rate distributed acoustic sensing (DAS), made available by the unique designs of certain interrogator units, acquires seismic data that are theoretically equivalent to the along-fiber particle velocity motion recorded by geophones for scenarios involving elastic ground-fiber coupling. While near-elastic coupling can be achieved in cemented downhole installations, it is less obvious how to do so in lower-cost horizontal deployments. This investigation addresses this challenge by installing and freezing fiber in shallow backfilled trenches (to 0.1 m depth) to achieve improved coupling. This acquisition allows for a reinterpretation of processed deformation-rate DAS waveforms as a “filtered particle velocity” rather than the conventional strain-rate quantity. We present 1D and 2D filtering experiments that suggest 2D velocity-dip filtering can recover improved DAS data panels that exhibit clear surface and refracted arrivals. Data acquired on DAS fibers deployed in backfilled, frozen trenches were more repeatable over a day of acquisition compared to those acquired on a surface-deployed DAS fiber, which exhibited more significant amplitude variations and lower signal-to-noise ratios. These observations suggest that deploying fiber in backfilled, frozen trenches can help limit the impact of environmental factors that would adversely affect interpretations of time-lapse DAS observations.

## 1. Introduction

Distributed acoustic sensing (DAS) in permanent monitoring installations is increasingly being used for seismic investigations [1,2,3,4,5]. Permanent downhole fiber installations often achieve near-perfect elastic coupling with the surrounding earth (e.g., by cementing a fiber to the outside of casing), which enables some interrogator units (IUs) to acquire seismic data of a quality approaching the response of single-component geophones [6,7]. However, for lower-cost surface fiber deployments (e.g., fiber directly laid on the ground, passed through buried conduits, or placed in shallow trenches), it is less obvious how to effectively achieve elastic ground-fiber coupling and thereby avoid the associated loss in data quality. A related important question is how significant the deleterious effects of imperfect coupling are on DAS recordings for scenarios involving spatial and/or temporal variations in the ground-fiber coupling.

Despite the complexities involved in near-surface horizontal fiber installations, many successful DAS research examples using such deployments have emerged in the literature. For terrestrial deployments, Becker et al. presented a laboratory experiment that demonstrates the possibility of DAS-based earth-tide observations [8]. Fernández et al. showed the promising performance of low-frequency (<1 Hz) DAS recording when compared to high-quality broadband seismometers [9]. Spical et al. used the ambient recording from a horizontal DAS array deployed on Stanford University campus to calculate the horizontal over vertical (H/V) spectral ratio and compute interpretable near-surface imaging results [10]. Yuan et al. investigated Rayleigh waves excited by passing cars recorded on a roadside section of the Stanford DAS-2 array and constructed a pseudo-2D shear-wave velocity profile by integrating 1D inversions [11]. Fang et al. demonstrated the feasibility of using an existing horizontal DAS array for near-surface velocity monitoring by measuring strong time-lapse variations [12]. Lindsey et al. analyzed and calibrated the sub-1 Hz DAS instrument response using co-located broadband seismometer records as the reference of true ground motion [13]. Shragge et al. presented the results from a low-frequency DAS experiment that uses surface waves to constrain the shear-wave velocity profile to 0.5 km depth [14].

There is also a growing number of examples that use optical fiber deployed in marine seafloor environments. For example, Williams et al. and Sladen et al. successfully recorded ocean microseism energy and detected regional earthquakes using ocean-bottom fiber arrays with onshore DAS IUs [15,16]. Lindsey et al. presented observations from four days of recording on an ocean-bottom “dark fiber” array and detected various signals such as minor earthquakes, primary and secondary microseisms, and sediment transport due to storm action [17]. Jousset et al. demonstrated the application of 15 km of dark telecommunication fiber deployed on the Reykjanes Peninsula, Southwest Iceland to record and process high-resolution seismic waveforms that showed features such as normal faulting and volcanic dykes with unprecedented resolution [18]. Cheng et al. constructed a near-seafloor velocity model and developed improved constraints on shallow submarine faults by inverting multimodal dispersion curves obtained from ambient DAS records acquired on 20 km of ocean-bottom cable [19]. Ide et al. observed many earthquakes using a submarine cable located near the Nankai subduction zone [20]. Finally, Lindsey et al. provided a review of the increasing number of long-term monitoring experiments conducted in US national labs and universities [21].

An interesting observation is that non-elastic coupling is not commonly discussed in the aforementioned DAS literature. We postulate that this is because most IU designs either natively acquire DAS data in strain or strain-rate format that necessitates assuming a gauge length (GL) defined in hardware or applying a GL post-acquisition through digital processing to generate interpretable waveforms. Because applying a GL generally involves introducing a 1D spatial filter, this can adversely affect the wavenumber spectra of DAS records by introducing spectral notches (i.e., zeros) as well as variable spectral weighting factors [22]. While there are digital signal processing approaches that could be used to mitigate these effects, they are also susceptible to boosting unwanted signal or noise when aiming to recover “lost” or down-weighted spectral information.

This work presented here similarly examines DAS data acquired on a near-surface horizontal fiber array; however, we use a Terra15 Treble DAS IU with a novel optical measurement design that measures a “deformation-rate” quantity [23]. Under ideal elastic fiber-ground coupling conditions, this deformation-rate measurement acquires data that are theoretically equivalent to the single component of the ground particle-velocity vector recorded on a fiber segment oriented in the fiber axis direction. This proprietary IU design leads to a particle-velocity-equivalent quantity $\tilde{v}\left(x,t\right)$ that effectively represents the integral of the strain rate $\dot{\varepsilon }$ from the interrogation point (at u=0) to point u=x on the fiber

(1) $$\tilde{v}\left(x,t\right)=\int_{0}^{x}\dot{\varepsilon }\left(u,t\right)\;du,$$

where *t* is time, *u* is an auxiliary spatial integration variable, and the tilde symbol on $\tilde{v}$ emphasizes that the measured quantity is only equivalent to the true particle velocity of ground motion when the elastic fiber-ground coupling conditions are satisfied.

To demonstrate this near equivalence, the authors of [24] acquired DAS data on a Treble IU in deformation-rate format on a completed downhole fiber installation to show the near identicalness to the ground motion recorded on a co-located borehole geophone array. Their observations indicate that when combined with a deployed fiber elastically coupled to the borehole casing, Treble IU acquisition can achieve a geophone-like cosθ sensitivity pattern where θ is the incidence angle [25] compared to the conventional $\cos^{2}\theta$ relationship of strain-rate DAS measurements [26]. Consequently, the strain-rate DAS measurement suppresses waves arriving at greater incident angles, resulting, e.g., in limited sensitivity to far-offset P-wave arrivals in vertical seismic profiling (VSP) experiments. This leads to a decreased usable angular bandwidth (i.e., $\cos^{2}60^{\circ }=0.25$) [27]. Comparatively, the deformation-rate format allows a broader usable angular bandwidth (i.e., $\cos60^{\circ }=0.5$). Importantly, Sidenko et al. demonstrated that this improved angular bandwidth can be achieved without post-processing spatial-derivative filtering to recover high-quality particle-velocity-equivalent DAS signals in the deformation-rate format, which forestalled introducing the associated adverse filtering effects (e.g., spectral notches) commonly present in DAS strain-rate observations [24].

Motivated by these observations, this study investigates whether the advantages of the Treble IU in the native deformation-rate (i.e., particle-velocity-equivalent) acquisition format highlighted by improved angular bandwidth can be realized for superficial as opposed to downhole fiber installations, and whether different 1D and 2D filtering operations can be applied to data acquired in this format to improve the signal-to-noise ratio. Our initial deployment experiments involving imperfect elastic fiber-ground coupling scenarios (e.g., draped on the surface, deployment in conduits) acquired DAS data which exhibited low-wavenumber noise that accumulated along the length of the fiber. Unfortunately, this noise needed to be handled through post-acquisition spatial filtering operations (e.g., applying a first-derivative filter with an assumed GL) and thus offered no improvement over standard strain-rate acquisition. These initial experiments underscored the importance of elastic fiber-ground coupling when using the Treble IU for the purpose of deformation-rate DAS data acquisition.

Aiming to achieve a horizontal DAS deployment scenario that approaches near-elastic coupling, we report the findings from an experiment that used an alternate approach to establishing coupling—freezing the fiber to the ground. We describe a small-scale investigation where we deployed three parallel fiber segments of 120 m total length in a shallow trench in the frozen earth that was watered down and left to freeze in the ground overnight. Over the following day, we acquired repeat sledgehammer shots as the outside air temperature reached 6.5 °C mid-afternoon and then fell to −6.5 °C by mid-evening. In addition to a fiber–soil coupling improvement, we expect the trenched-in fiber to be better insulated from the air temperature fluctuations than the surface-deployed fiber section.

We begin by providing additional detail about the interrogator design and by describing the data acquisition, including the experimental setup to enhance the fiber–ground coupling and the presentation of raw data results. Due to unwanted residual signals in the dataset, we then discuss the 1D and 2D filtering strategies used to improve the signal-to-noise levels of the filtered DAS data panels. We then show the processed DAS data that verify the efficacy of the proposed filtering method and compare the shot gather with the data recorded from the conventional vertical-component geophones. A brief discussion section examines the amplitude fluctuations noted in the surface-deployed fiber and cautions for DAS data interpretation when acquiring data over calendar time. The last section summarizes the effects of enhanced coupling on the frozen trench and filter designs on particle-velocity DAS recording.

## 2. DAS Data Acquisition

To investigate the role that frozen ground can play in the ground–fiber coupling, we selected suitable test dates (15–16 February 2021) when the weather forecast for the investigated location (Arvada, CO, USA) predicted that the air temperature would remain well below freezing on the first day, rise above 0 °C by mid-morning on the second day, reach 6 °C by mid-afternoon, and then return below 0 °C by the early evening. Our deployment involved in-ground trenching of 120 m of military-grade single-mode fiber, tactical tight-buffered cable with aramid strength members, and a polyurethane jacket. We first trenched a 35 m section of fiber into the ground at approximately 10 cm depth (Section A), covered it with soil, compacted the soil by hand with a tamper tool, and then thoroughly watered it down. We then looped the fiber back along the same trench at approximately 5 cm depth (Section B), again covering the fiber, compacting the soil, and watering it down. Finally, we deployed the remaining fiber directly on the surface (Section C). The three sections allowed us to examine coupling effects at different depths with likely variable sensitivity to air-temperature fluctuations.

**Figure 1:**
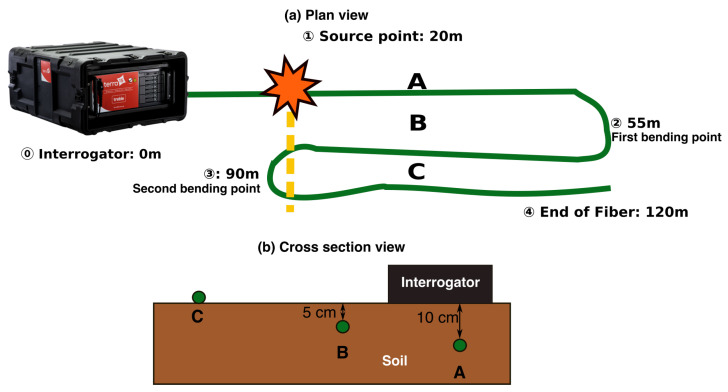
Schematic drawing of DAS acquisition geometry in plan (**a**) and cross-sectional (**b**) view. Sections A and B correspond to the trenched fiber locations at approximately 10 cm and 5 cm depth, respectively (see lower panel). The fiber in Section C is laid on top of the ground. In this layout, sections A–C respectively are between 20–55 m, 55–90 m, and 90–120 m in the along-fiber distance. The orange star and yellow dashed line indicates the source point at 20 m.

**Figure 2:**
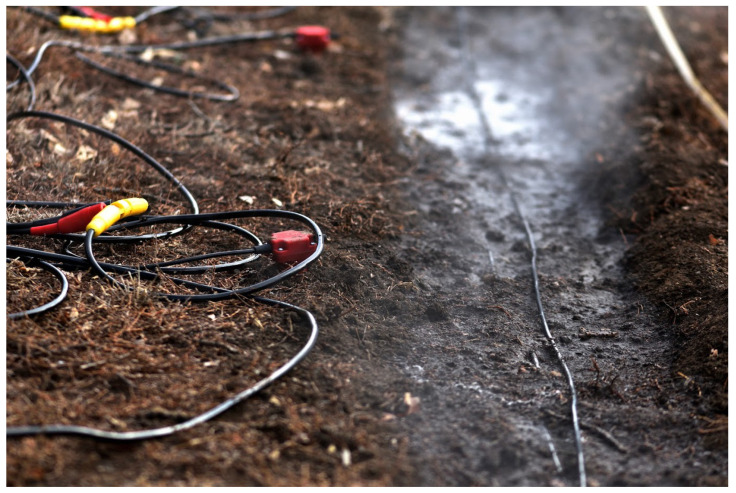
Fiber on Sections B and C with the deployed vertical geophones to the left. The steam is from water poured to freeze the fiber to the ground and improve the ground coupling. (Photo credit: I. Pawelec).

Figure 1a,b respectively show the overall fiber deployment geometry in both plan and cross-sectional view with the following points: (1) the interrogator is housed in a garage at point 🄋 at 0 m distance; (2) the source point is located ➀ at 20 m; (3) Section A runs between 20–55 m up to the turnaround point at ➁; (4) Section B runs between 55–90 m back to the turnaround point at ➂; and (5) Section C runs between 90–120 m up to the end of the fiber at ➃. Figure 2 depicts the installed fiber a few minutes after watering down the section. For comparison purposes, we installed vertical component geophones 0.3 m from the fiber as a baseline for comparison. (Unfortunately, horizontal geophones that would have offered a better comparison were not available at the time of the experiment).

The DAS IU used for the seismic acquisition was a circa mid-2020 Treble IU developed by Terra15 Technologies Pty Ltd of Perth, Australia. The phase-based system has a proprietary optical design constructed to eliminate amplitude and polarization fading [23]. The Treble IU can acquire DAS seismic data either in native deformation- or strain-rate format with the gauge length (GL) modifiable through post-processing. The IU measurement type and properties depend on the choice of the interference pair of optical signals. The Treble IU generates two pulses at a fixed time interval, and then correlates the returned time-delayed first pulse and optically delayed second pulse to natively measure a deformation-rate rather than a more typical strain-rate quantity. For more details on the optical process, we refer readers to the patent document of [23].

We acquired roughly 3.0 Tb of DAS data in continuous mode over the two-day experiment in the deformation-rate format at 0.038 ms and 0.8 m temporal and spatial sampling intervals, respectively. To test the temporal variability of ground-fiber coupling, we used a sledgehammer and metal plate as an energy source and generated shots at approximately 30-min intervals for 12 h from 9:00 a.m. to 9:00 p.m. Because of the fiber deployment pattern (see Figure 1a), the shot point was observed simultaneously at three effective shot locations on Sections A–C: 20 m, 90 m (back up the fiber), and 90 m (again down the fiber) from the IU, respectively.

The first processing step involved window selection where we used the noted shot times to extract 60.0 s data streams of approximately 1.0 Gb size. Because the frequencies of interest from the sledgehammer shots were lower than 150 Hz, we low-pass-filtered the extracted sections with a 150 Hz cutoff and subsampled the extracted shot windows to a more manageable data volume. We then corrected the polarity of the data acquired on Section B to compensate for the reversed fiber direction with respect to the shot location.

**Figure 3:**
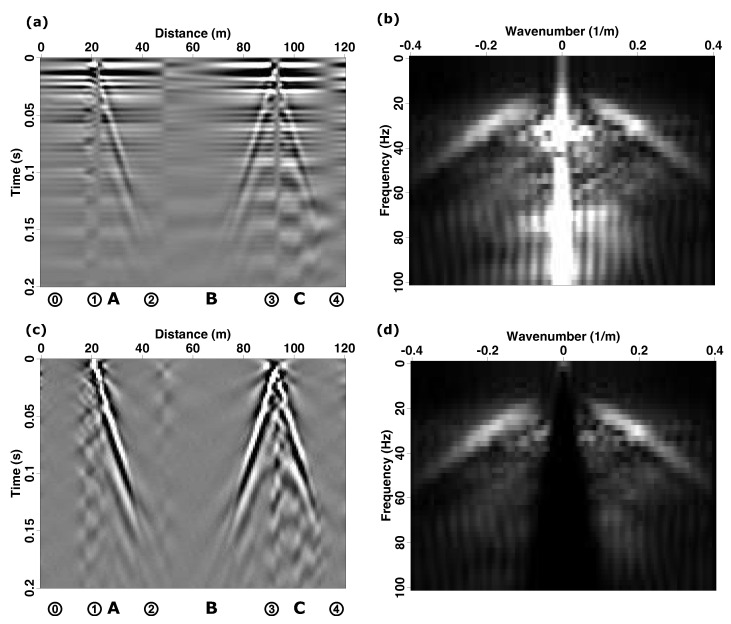
(**a**) Velocity data for a hammer shot recorded at 9:00 pm after applying the polarity correction to Section B. Note that the record is contaminated with horizontal noise that has a moveout too fast to be a seismic event. (**b**) The associated frequency–wavenumber f−k plot where the vertical “washboard” striping is due to the presence of the horizontal unwanted but repeated signals observed in (**a**). (**c**) 2D dip-filtered velocity data for the same shot in (**a**) that now shows refracted-wave arrivals. (**d**) The f−k spectra for the filtered shot record shown in (**c**).

Figure 3a presents a shot gather recorded at 9:00 p.m. in the deformation-rate data acquisition format of the Treble IU. Surface-wave arrivals are clearly identifiable in all three fiber sections. Although high-amplitude horizontal arrivals are observed in the raw data, the corresponding moveouts are too fast to be a passing seismic wave disturbance. However, these arrivals are repeatable and thus represent coherent “unwanted signals” that should be removed through signal processing. Figure 3b corresponds to a frequency–wavenumber (f−k) spectrum of the shot gather from Figure 3a. The identified coherent noise source maps to the strong low-wavenumber components observed as two lobes with between 20–60 Hz as well as the vertical “washboard” pattern corresponding to the quasi-horizontal signals in the time–space (t−x) panel in Figure 3a.

We observe that the distortion-rate recording from section A enclosed in frozen soil captures traveling surface waves well throughout the day, which is consistent with our expectations of improved fiber–soil coupling. Despite the clear arrivals presented in the distortion-rate data panel, low-wavenumber noise persists that reduces the quality of the data and ensuing processing results. Herein, we suggest that one can apply low-cut filtering such as a 1D gradient operator to eliminate low-wavenumber noise persisting in the deformation-rate data. Thus, we next examine the efficacy of several 1D and 2D filter operators to find a judicious approach for processing low-wavenumber-contaminated deformation-rate waveforms.

## 3. DAS Data Filtering Procedure

The next conventional processing step (though not necessarily required for this dataset) would be to calculate strain-rate data from the deformation-rate measurement by applying some variation of a spatial filtering operator (e.g., a GL or first-derivative filter). To illustrate the associated effects of undertaking such a filtering operation, we examine the effects of applying two classes of filtering operations: (1) spatial 1D finite impulse response (FIR) and infinite impulse response (IIR) filters of different orders of accuracy, and (2) 2D dip-velocity filtering.

### 3.1. Spatial 1D Filtering

The first approach in the 1D filter class is to apply a conventional GL filter of length $L_{G}$ through

(2) $${\dot{\varepsilon }}_{E}\left(x,t|L_{G}\right)\approx \frac{\tilde{v}\left(x+L_{G}/2,t\right)-\tilde{v}\left(x-L_{G}/2,t\right)}{L_{G}},$$

where ${\dot{\varepsilon }}_{E}$ is the estimated along-fiber strain rate.

The second approach in the 1D filter class is to use a 12th-order Taylor-series finite-difference (FD) approximation of the analytic first-derivative operator,

(3) $$\dot{\varepsilon_{E}}\left(x,t|c_{k}\right)\approx \frac{1}{\Delta x}\sum_{k=1}^{6}c_{k}\left(v(x+k\Delta x,t)-v(x-k\Delta x,t)\right),$$

where $c_{k}=\left[23760,-7425,2200,-495,-72,5\right]/27720$ are the corresponding FD coefficients and Δx is the spatial sampling interval along the fiber (a user-defined parameter).

**Figure 4:**
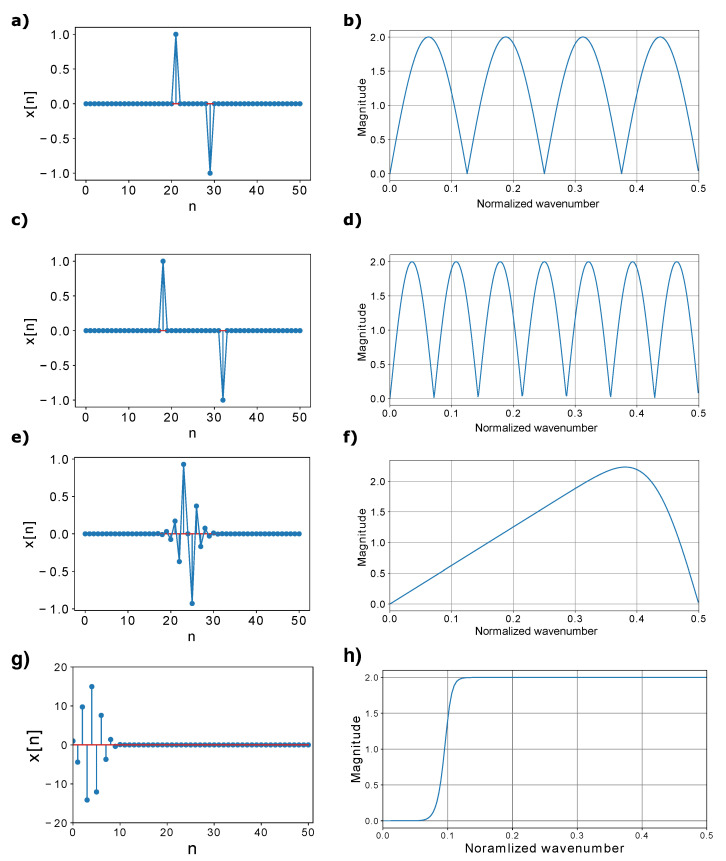
Impulse response filters with their associated magnitude spectra. (**a**) $L_{G}$ = 8-unit FIR filter. (**b**) Magnitude spectrum of the filter in (**a**). (**c**) $L_{G}$ = 14-unit FIR filter. (**d**) Magnitude spectrum of the filter in (**c**). (**e**) 12th-order FD first-derivative FIR approximation. (**f**) Magnitude spectrum of the filter in (**e**). (**g**) Filter coefficient of high-pass Butterworth IIR filter. (**h**) Corresponding magnitude spectrum of the filter in (**g**).

To illustrate the behavior of the different filtering approaches, the left and right columns of the upper three rows of Figure 4 present three different FIR filters along with their associated spectral responses. Overall, we observe that the different filtering approximations lead to very different strain-rate data results. In particular, a shorter GL introduces stronger near-zero wavenumber filtering (Figure 4a,b), while a longer GL exhibits reduced near-zero filtering but introduces additional spectral notches (Figure 4c,d). Conversely, the 12th-order spatial first-derivative filter (Figure 4e,f) does not introduce the notches except at DC and the normalized Nyquist wavenumber (i.e., 0.5 m${}^{-1}$). This filtering approach also imparts a more accurate representation of the expected |k| spectral magnitude of the first-derivative operator that leads to a linear increase in the weighting factor with increasing wavenumbers until reaching the effective approximation limit at about 70% of the Nyquist wavenumber.

**Figure 5:**
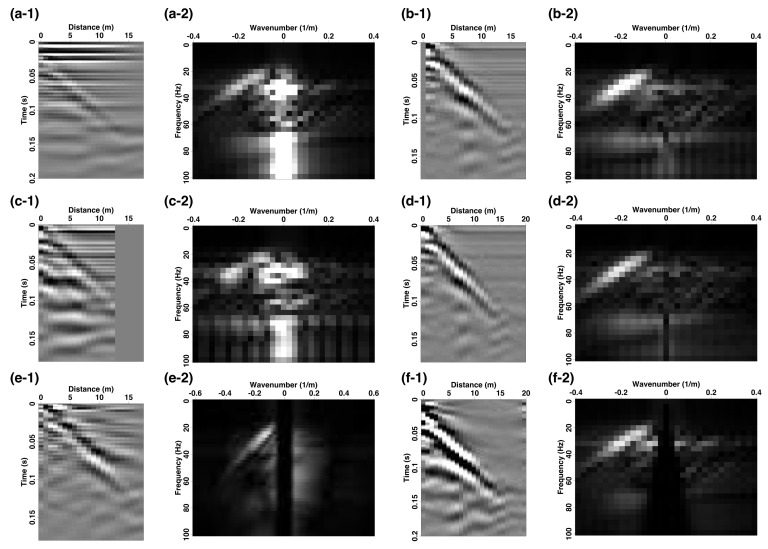
Data and f−k spectra for a shot gather recorded on Section A. (**a-1**) Raw deformation-rate data and (**a-2**) the associated f−k spectra. Strain-rate data with (**b-1**) $L_{G}$ = 0.8 m and (**b-2**) the associated f−k spectra. Strain-rate data with (**c-1**) $L_{G}$ = 6.4 m and (**c-2**) the associated f−k spectra. (**d-1**) Strain-rate data after applying a 12th-order accurate numerical first-derivative filter as shown in Figure 4e and (**d-2**) the associated f−k spectra. (**e-1**) 1D low-cut Butterworth filter and (**e-2**) the associated f−k spectra. (**f-1**) 2D velocity-dip filtering and (**f-2**) the associated f−k spectra.

Figure 5 illustrates the denoising benefits of applying 1D filtering to obtain a strain-rate panel estimate for data windowed out for Section A. Figure 5a-1,a-2 present the raw deformation-rate data of the windowed subset as well as the corresponding f−k spectra, respectively. We next apply the gauge-length filter with $L_{G}=\Delta x=0.8$ m, equivalent to the spatial sampling interval. The low-order 1D spatial-derivative filter acts as a low-cut filter that through the strain-rate conversion removes the strong low-wavenumber noise in Figure 5b-1,b-2, but also significantly upweights signal and noise at higher wavenumbers. Figure 5c-1,c-2 show the results of applying a GL filter of length 6.4 m (i.e., $L_{G}=8\Delta x$), which has done a poorer job of removing the unwanted horizontal signal and clearly introduces notches that adversely affect the observed f−k spectrum (Figure 5c-2) and is consistent with the frequency response shown in Figure 4d. Figure 5d-1 presents the data panel filtered by the 12th-order FD approximation (Equation (3)) while Figure 5d-2 presents the associated f−k spectra. We observe that these higher-order filtering operations do a better job than the lower-order GL counterparts, as indicated by the improved quality of the unwanted horizontal signal removal and the lack of notches in the f−k spectra.

### 3.2. 1D High-Pass Wavenumber Filtering

Although applying a GL (or low-order first-derivative) filter to calculate a strain-rate quantity is by now a standard technique, we point out that the deformation rate (i.e., particle-velocity equivalent) $\tilde{v}$ measured in the Treble IU allows for a different interpretation of the filtering process—namely, the purpose of applying a filtering operator is to denoise $\tilde{v}$ rather than transform $\tilde{v}$ into a derived strain-rate quantity $\dot{\varepsilon }$. To illustrate this, we examine the application of an IIR filter to the same data as used in the 1D derivative filtering above. To implement the IIR filter, we used the scipy.signal.butter package [28], which provides both digital and analog Nth-order Butterworth filters. Figure 4g,h illustrate one example of a high-pass Butterworth IIR filter generated using numerator coefficients of a=[1.0,4.4,9.7,14.1,14.9,12.1,7.6,3.7,1.4,0.4,0.07,0.06] and denominator coefficients of b=[1,0,0,0,0,0,0,0,0,0,0,0]. The resulting frequency response shows that one can effectively remove low-wavenumber noise while preserving the remaining signals. We apply the high-pass Butterworth filter to the same windowed subset (Figure 5a-1). Figure 5e-1,e-2 respectively show the resulting data panel and the corresponding f−k spectrum, which clearly displays the removal of low-wavenumber noise. However, the data panel also exhibits remnants of horizontal noise around 0.0 s to 0.05 s, which motivates the exploration of further filter designs.

### 3.3. 2D Frequency–Wavenumber (f−k) Filtering

The above 1D high-pass filtering approach conceptually can be extended into a 2D operation through the use of velocity-dip filtering [29], which has been used for decades in seismic data processing to remove the types of unwanted coherent signal observed in Figure 3a and Figure 5a-1,a-2. Coherent linear events, such as guided waves and ground roll, are straightforward to identify by their associated dip and remove in the f−k domain (i.e., after applying a 2D Fourier transform). After locating an event in the f−k domain, one can apply a 2D multiplicative “fan-like” mute (with tapered edges) to reject the identified unwanted data components. One then recovers the filtered t−x domain section by applying an inverse 2D Fourier transform. The 2D velocity-dip filter is straightforward to implement, has few tunable parameters, and leads to high-quality filtered output when the desired signal and unwanted signal/noise dip spectra are clearly separated as they are in this dataset. Following such an approach again allows for the resulting panel to be considered as a “filtered $\tilde{v}$” panel rather than an ${\dot{\varepsilon }}_{E}$ panel estimate.

Figure 5f-1,f-2 illustrate the benefits of applying the dip-filter approach to this dataset. The fan-like pattern observed in the f−k spectra, corresponding to a passband for waves traveling horizontally at apparent velocities ($V_{app}=df/dk$) between 0.1 km/s and 1.5 km/s, has removed the near-zero wavenumber energy without introducing notches or significant upweighting of high-wavenumber components. Applying the 2D filtering approach to the entire fiber section also highlights its benefit, as shown in Figure 3c. It presents a 2D dip-filtered version of the raw panel presented in Figure 3a. The resulting panel is largely free of the unwanted horizontal signals making the weak refracted waves more apparent. Figure 3d presents the f−k spectra associated with Figure 3c. The dip-filter reject band about the DC wavenumber is quite apparent; however, the remaining components of the f−k spectra are untouched. Thus, 2D velocity-dip filtering represents our preferred approach for this dataset, though we point out that this approach is likely less useful when applied to datasets with poorer ground-fiber coupling.

## 4. Discussion

Having now specified our preferred form of 2D filtering and interpretation, we now turn to making two additional sets of comparisons: (1) how well do the filtered DAS panels match geophone data? (2) How repeatable were the shot gathers acquired on the DAS system over the acquisition period?

**Figure 6:**
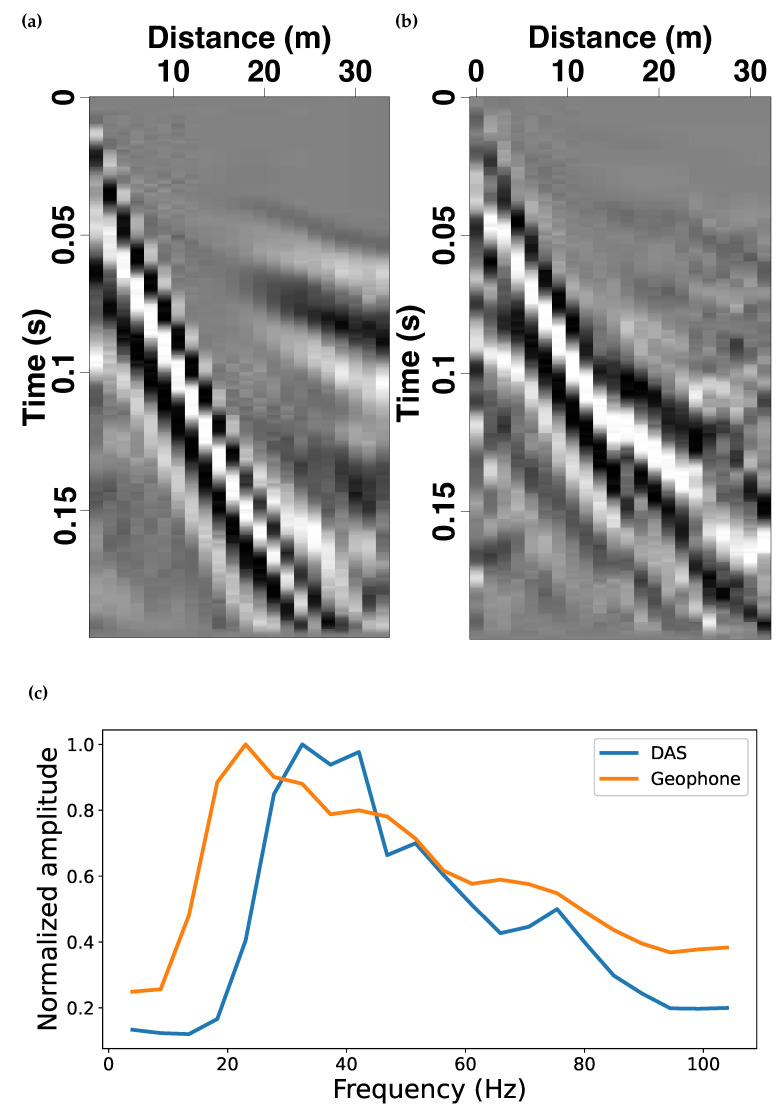
Representative shot gathers recorded on (**a**) vertical-component geophones and (**b**) DAS fiber array (after 2D velocity-dip filtered). (**c**) Normalized magnitude spectrum of the two shot gathers presented in (**a**,**b**).

We first present a comparison between the recordings acquired on the horizontal DAS fiber with those made on the adjacent vertical-component geophones. Figure 6a,b present geophone- and DAS-acquired shot gathers, respectively. The surface wave recorded from both sensors displays comparable arrival phases and apparent moveout velocities. Figure 6c presents the stacked magnitude spectra for both records. We observe that the dominant frequency band of the DAS and geophone records largely overlap; however, the geophone data are shifted to lower frequency and appear to have a somewhat broader response.

**Figure 7:**
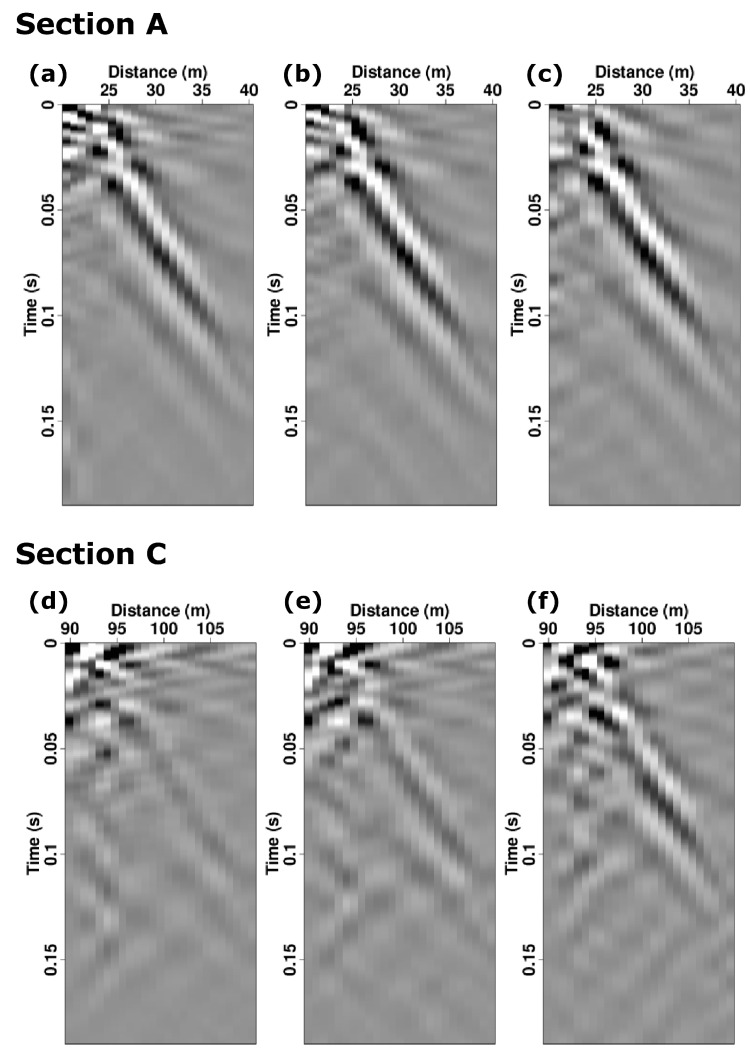
Repeat deformation-rate DAS shot gathers recorded on the frozen, backfilled trenched fiber Section A (upper panels) and the surface-deployed fiber Section C (lower panels). (**a**,**d**) 3:00 p.m. (**b**,**e**) 5:30 p.m. (**c**,**f**) 9:00 p.m.

To examine the repeatability of the DAS shot gathers and the potential for diurnal variability, we evaluate three shots acquired at Sections A and C acquired at three different times of the day. Figure 7a–c presents three different shot gathers recorded on fiber section A at 3:00 p.m., 5:30 p.m., and 9:00 p.m., respectively. These three shot gathers display consistent moveouts, waveforms, and amplitudes, which suggests that the shot records are largely repeatable throughout the time of the investigation. Figure 7d–f presents the same three shot gathers as presented in Figure 7a–c; however, these were recorded Section C of the fiber deployed on the surface. Unlike in the top three panels, the bottom three panels exhibit significant amplitude and phase variations. These observations suggest that trenching and freezing in fiber improves not just ground-fiber coupling, but also measurement repeatability.

Finally, the significant variability of the shot gathers presented in Section C are unlikely to be due to significant time-lapse variations of the bulk properties of the near-surface earth model. Rather, a more likely explanation is that as the air temperature drops from above to below freezing, one should expect refreezing of moisture in the soil and likely improved adherence of the soil to the fiber. Accordingly, the shot gather acquired at 9:00 p.m. (Figure 7f) exhibits stronger amplitudes and higher signal-to-noise ratio compared to those acquired at 3:00 p.m. and 5:30 p.m. (Figure 7d,e). Thus, we stress that interpreting the amplitudes (and potentially phases) of DAS data prior to removing or controlling for external environmental factors requires due caution. We encourage further examination using a hybrid system that measures strain and temperature simultaneously, such as a combined DAS and DTS (distributed temperature sensing) system or DVTS (distributed vibration and temperature sensing) [30]. Such hybrid systems already provide solutions in various fields including oil and gas, mining, and civil engineering. For our work, future hybrid measurement will help to clarify the amplitude changes or help design a filter for the decoupling temperature effect.

## 5. Conclusions

We present an experiment that evaluates the Terra15 Treble IU DAS deformation-rate format recorded on a fiber array deployed at variable freezing conditions and trenching depths. We propose a suitable filtering methodology compared to the existing methods such as strain-rate transformation and low-cut filters. The recorded deformation-rate data showed coherent surface-wave arrivals; however, refracted arrivals only clearly appeared after applying our preferred 2D dip-velocity filtering approach. Therefore, we expect enhanced angular bandwidth from 2D dip-filtered deformation-rate data compared to that of the strain rate. The trenched and frozen-in fiber provided sufficient coupling to record usable deformation-rate data throughout the day; however, data recorded on the fiber section simply laid on the ground show decreased overall quality and exhibit time-dependent effects due to the variable ground-fiber coupling likely introduced by air-temperature fluctuations about 0 °C. Our results suggest that trenching and freezing can provide improved ground-fiber coupling for recording in the Treble IU deformation-rate format. The benefit of enhanced coupled through freezing is likely similar for other IUs, which should encourage future DAS experiments such as installations on lakes, glaciers, and snowpack in sub-0 °C environments.

## Data Availability

Data were recorded at Perth by Terra15. Derived data supporting the findings of this study currently are not available due to proprietary considerations. Processed data sets are available in DRYAD open repository: (https://datadryad.org/stash/share/-Yq1WGlQmCGMM20yFELahjZtLHtHyeOctBuRyDnzH38) (accessed on 24 October 2022).
